# Is It Structure or Environment?*Read before the Odontological Society of Chicago, February 5, 1918, and reprinted from the May *Dental Review*.

**Published:** 1918-05

**Authors:** C. N. Johnson

**Affiliations:** Chicago, Ill.


### IS IT STRUCTURE OR ENVIRONMENT?*

BY C. N. JOHNSON, M.A., L.D.S., D.D.S., CHICAGO, ILL.

It will be noted that this essay starts with a query, and it may be possible that it will end with one. The question relates to the difference of opinion still existing among the members of the profession as to whether the significant factor in dental caries is the character of the teeth themselves or the conditions which surround the teeth. In other words is it structure or environment? Much may be said for and against either proposition, and it is well that the profession study this matter more and more carefully till a final solution is reached.

This study must inseparably be linked with the manifestations of susceptibility and immunity in the mouths of our patients. We know from observation that the mouths of some individuals are very susceptible to dental decay, while others are practically immune. We also know that there is a great variation in the degree of susceptibility manifested at different periods in the same mouth. The question is, does this variation relate to differences in the tooth structure, or is it due to differences in the environment which surrounds the teeth?

For a long time the opinion was quite prevalent that the varying manifestations of dental decay were due to a

*Read before the Odontological Society of Chicago, February 5, 1918, and reprinted from the May *Dental Review.*

resistance or a lack of resistance on the part of the tooth tissue; that some teeth were "harder" than others and resisted the active agents of decay, while others were "softer," and thus succumbed. For the time being the fact seemed to be overlooked that in the various chemical analyses that were made of the teeth by different men the percentages of organic and inorganic material ran along with astonishing uniformity, that there is very little variation in the chemical constituents of the teeth of different individuals; and moreover, that what little variation there may be seems to have almost no relation to the susceptibility or immunity of any mouth.

This question of "hard" and "soft" teeth is a very natural one, and it is easily explained. Dentists in operating on the teeth discovered that in some instances the tooth tissue would break down quite easily under cutting instruments, while in others it was stoutly resistant. The natural inference was that in the one instance the teeth were soft and in the other they were hard. This idea planted itself in the minds of dentists, and from them it found lodgment in the minds of patients. In its practical bearing it proved a very disastrous theory to promulgate. It gave the impression to many people that their teeth were too soft to be saved by filling, and they were accordingly frequently condemned to the forceps, when a conscientious effort on the part of the dentist and patient would have saved them.

A careful observation of the phenomenon presented by the difference in the behavior of teeth under cutting instruments will reveal the fact that this difference is confined for the most part to the enamel, and that it does not relate to any variation in the chemical constituents of the teeth, but to the mere mechanical fact of the difference in the arrangement of the enamel rods. As everyone in the profession knows, the enamel rods stand with one end resting on the dentin and from this radiating out toward the external surface of the crown of the tooth. It is the particular manner of this radiation that controls the resistance or lack of resistance of the enamel to cutting instruments. In some instances the rods radiate in a straight, regular, and almost parallel manner; while in others they pursue a wavy irregular course; this difference in the arrangement of the rods making the difference between enamel which cleaves easily and that which resists the instrument. It is well illustrated by the homely example of the difference between splitting straight-grained maple and bird's-eye maple. Every boy brought up in the country knows what a joy it is to split straight maple as compared with the grief attached to the attempt to split bird's-eye.

In breaking down enamel overhanging a cavity with a chisel it will be found that the enamel cleaves readily in line with the rods, but it is almost impossible to break the rods across. This proves that the strength of the enamel rests in the rods, and not in the cement substance which holds the rods together. In straight-grained enamel the chisel may be so applied that the enamel is cleaved away along the line of the rods, thus separating them where they are cemented together without breaking off the rods themselves; but in the wavy variety this can not be done. In order to break down wavy enamel it is necessary to fracture across many of the rods and this is what chiefly constitutes the difficulty in cutting such enamel. But the significant fact remains that if a chemical analysis is made of the two varieties of enamel it will be found that they are practically the same, and a matter of still greater significance is apparent in the fact that so far as susceptibility to decay is concerned the one enamel is as susceptible as the other. That is one enamel will take on the beginnings of decay as readily as the other, provided the environment is the same. Of course there may be a slight variation in the rapidity with which caries penetrates the two kinds of enamel. Manifestly the acid of decay can not travel so rapidly down along the cement substance of enamel in which the rods are wavy and irregular as it can beside rods which are straight, but this does not alter the general fact that in a susceptible mouth both enamels are attacked with apparently equal facility. In other words there was never yet enamel laid down in the human mouth that would resist decay if the conditions surrounding the tooth were favorable to the development of caries, while there are assuredly countless numbers of teeth in which the enamel would be found to break down easily if cut into, and which seem thin and frail in structure, but which remain free from decay for a lifetime.

Another consideration relates to a fact already referred to—that we note in the same mouth great differences in the tendency to decay of the teeth at different periods. All of us have noted this, a typical instance of which is a child with rapidly developing caries up to a certain age, when, if proper attention is given the teeth, there seems to be a cessation of the carious process and the establishment of immunity. Then again, let this same patient later in life undergo some prolonged physical or mental tension, and the teeth are likely to begin to melt away with caries almost like snow before the summer sun. What is the reason? Is it because the structure of the teeth has changed so that at the one period they are more vulnerable to attack than at another? Do the teeth grow hard and resistant at one time, and soft and susceptible at another time? Is it not a fact that of all the tissues of the body the teeth are the least subject to change of any? In truth are we not told that the teeth are the only organs of the human economy which are not constantly being torn down and built up in the physiological processes of nature; that the teeth when once laid down and calcified in the jaw remain to all intents and purposes the same through life? Cut a piece from a muscle and nature will fill in the wound; break a bone and nature will proliferate new cells and mend the break, take a section from a nerve trunk and nature will restore that nerve to an incredible length, as has so frequently happened to the discomfiture of the surgeon and disappointment of the patient in operations for neuralgia.

But what shall we say of teeth? Fracture a cusp through the enamel and see if nature will restore that cusp. Not in a lifetime. Nature seems to have appointed the teeth as the one and only stable structure, not amenable to the same laws of constant change as are the other tissues. All of which points to the fact that when we see these varying manifestations of susceptibility and immunity to dental caries we are not logical in attributing them to changes taking place in the structure of the teeth themselves, but rather to changes occurring in the conditions surrounding the teeth—in other words to environment.

And this must be looked upon as a most fortunate circumstance. If decay of the teeth were due to the character of the tooth tissue the dentist would be helpless in the face of it. As has just been seen, we can not hope to change the structure of the teeth themselves, but we may hope to change the environment. And in this one direction lies the future promise of limiting, and eventually of preventing, dental caries.

By the foregoing it is not intended to convey the impression that the tooth remains wholly passive under the carious process, nor that there are not structural defects in the teeth which largely influence the formation of cavities. When a cavity begins in a tooth from whatever cause, and the dentinal fibrils are reached, there is an effort on the part of the pulp to protect itself by a deposition of calcareous matter in the pulp chamber at the point where the decay threatens; and if the approach toward the pulp is sufficiently slow it will be found that the pulp will recede indefinitely, all the while throwing out a deposit of secondary dentin to fill the pulp chamber at this point. This has been noted frequently in cases where the tooth has been worn down by mechanical abrasion, leaving the original outline of the pulp chamber clearly marked and filled with new tissue. This proves that so far as the pulp is concerned it is not inactive under the approach of caries, and to this extent it may be said to have some resistive function against decay, but it must be noted that this is confined to the pulp, and it is not operative until decay has actually started. It has nothing whatever to do with enamel nor to any resistance against the inauguration of caries in this tissue.

Then again we have structural defects in the tooth tissue due to faulty development, and in these defects we frequently find the beginnings of decay. Pits and fissures in the enamel left by a failure of the islands of calcification to coalesce and form a continuous covering to the tooth are often the seat of caries, and in this light the tooth structure may be said to be at fault. But two very significant considerations are worthy of note in this connection. We find that there are many mouths in which there is no decay even when these pits and fissures are present in the enamel, and not only this, but in susceptible mouths we see decay occurring in surfaces of the teeth where there are no pits or fissures and no structural defects of any kind. We are all familiar with the fact that the proximal surfaces of the teeth are looked upon as the most vulnerable to attack of any, and we know that at this point the enamel is as perfectly laid down as any place on the tooth.

In the light of all this it must be obvious to anyone that the significant factor in dental caries lies in the environment in which the teeth are placed rather than in the inherent quality of the tooth structure itself. This in reality is a most fortunate thing, because if ever we hope to control decay or to prevent it, we must do so through the medium of changing the environment rather than changing the teeth. As we have seen we can not change the teeth after they have once been erupted, though we may well hope to change the conditions which surround them. And all the virtue from oral prophylaxis so far as limiting dental caries is concerned comes from this very thing. Those enthusiasts who have proclaimed that by oral prophylaxis they have improved the tooth structure and rendered it more resistant against decay have merely polished the surface of the tooth and made it so smooth that it is thereby more readily kept clean. This is a very important function to perform, and it is deserving of all praise, but the profession should not delude itself as to what actually happens The polishing does not change the internal structure of the tooth—it merely places the surface in such a condition that external agents can not act upon it with the same facility. It also removes for the time all those influences of environment which tend to act upon the tooth—the gelatinous material under cover of which the microorganisms form their acid and attack the enamel. It takes away such mechanical irritants as deposits of all kinds, overhanging fillings, impinging edges of banded crowns, etc. All of these irritants tend to keep the surrounding tissues in an unhealthy condition and it is the change in these conditions around the teeth by which the greatest value of oral prophylaxis is expressed.

Unfortunately at the present time it is difficult to state in exact terms what the significant factor in dental caries really is. We know, as has already been intimated, that one mouth is susceptible and another immune, but we do not know what particular agent or influence makes the difference. Various men from time to time have suggested various elements as the deciding factor, but no one to date has given us a working theory by which we could unerringly demonstrate susceptibility or immunity in a mouth. Miller proved, of course, that decay was brought about by the presence of an acid produced by microorganisms, but microorganisms may be found in every mouth, immune as well as susceptible. There is something aside from the mere presence or absence of microorganisms which controls dental caries, and until we know just what this is we can not proceed definitely or logically toward the task of eliminating decay from the mouth.

This does not mean that we are helpless in the presence of decay, and limited only to the make-shift expedient of filling cavities after they have occurred. It is true that many in the profession are doing this very thing in a wholly perfunctory and routine way, looking no further than the mere fact of a cavity and a filling. But modern dentistry can offer something better than this. While the effort to administer drugs to change a susceptible mouth to an immune one has not met with the success which its advocates had hoped, yet there are other means by which we may proceed to bring this desirable consummation about. We can not stop the tendency to decay in all cases, but we can do so in many; and in every case we can, with the cooperation of the patient, work a very great improvement and keep down the decaying process to reasonable limits.

Resolved to its ultimate analysis it is merely a matter of cleanliness, and the intelligent practice of oral prophylaxis is the most effective means of bringing about cleanliness yet suggested to the profession. The judicial periodical removal of all gelatinous material from the teeth, the massage of the gums by the finger or tooth brush, the polishing of the teeth to keep the surfaces bright and glistening, and this kept up as a duty by the dentist and the patient will ultimately change the conditions in the mouth so that there will be a greatly lessened tendency to decay. This does not mean that it is justifiable for the dentist to promise the patient, as many are doing, that "a prophylactic treatment once a month will prevent decay;" nor does it imply that the term "prophylaxis" in this connection is a wholly correct one. It is even doubtful if this term should ever have been introduced, and used as the profession is using it today, because in many instances it is a misnomer. But the systematic plan of procedure as outlined under the head of oral prophylaxis presents to us at the present time the most promising means of limiting dental decay, and if this is true it comes very nearly answering the question at the head of this paper, "Is it structure or environment?"

Viewed in the light of what we now know, while the structure of the tooth may have some influence on the progress and rapidity of decay, the dominating factor in its incipiency is the environment in which the tooth is placed.

